# Diagnosing Integrated Clinical Performance: A Practical Educational Framework for High-Stakes Clinical Assessment

**DOI:** 10.7759/cureus.114316

**Published:** 2026-08-10

**Authors:** Zuhayer Ahmed, Tanvir Faisal Rakin, Zainab Kallow

**Affiliations:** 1 Endocrinology and Diabetes, Mersey and West Lancashire Teaching Hospitals NHS Trust, Liverpool, GBR; 2 Acute Internal Medicine, Mid Yorkshire Teaching NHS Trust, Wakefield, GBR; 3 Rheumatology, Northwick Park Hospital, London, GBR

**Keywords:** clinical skills practice, mrcp (uk) examinations, part 2 clinical examination (paces), postgraduate medical assessment, workplace-based learning

## Abstract

Introduction: High-stakes clinical performance examinations increasingly assess integrated clinical competence rather than isolated technical skills. The 2023 revision of the Membership of the Royal Colleges of Physicians of the United Kingdom (MRCP (UK)) Practical Assessment of Clinical Examination Skills 2023 (PACES23) exemplifies this shift by placing greater emphasis on communication, consultation, shared decision-making and patient safety alongside physical examination and clinical reasoning. Although relevant educational evidence exists across multiple domains, practical frameworks linking assessment demands with targeted workplace teaching remain limited.

Materials and methods: We conducted a structured narrative educational review integrating official PACES23 guidance with literature on clinical assessment, clinical reasoning, communication, feedback, deliberate practice, simulation and workplace learning. Through iterative interpretive synthesis, candidate and teacher behaviours were consolidated, mapped to the seven assessed PACES23 capabilities and organised into higher-order educational domains with associated teacher actions.

Results: We propose an integrated educational framework that couples a clinical enactment cycle (SEE → THINK → ACT) with a complementary learning cycle (PRACTISE → PERFORM → REFLECT). The first identifies where performance breaks down during clinical encounters, while the second guides targeted educational responses. The framework is operationalised through four educational domains and twelve evidence-informed strategies linking observable learner behaviours, educational mechanisms and practical teaching actions.

Conclusions: Using PACES23 as an illustrative context, this framework provides clinical teachers with a practical approach for diagnosing performance breakdown and delivering targeted workplace teaching and feedback. It shifts preparation from examination coaching towards longitudinal development of safe, integrated and patient-centred clinical performance.

## Introduction and background

Clinical performance examinations are used internationally to assess whether learners can integrate history taking, physical examination, clinical reasoning, communication and patient management into safe clinical practice. Although assessment formats differ across settings, they share a common educational purpose: evaluating integrated clinical performance rather than isolated knowledge or technical skills. This aligns with contemporary concepts of assessment that emphasise observable performance and integrated competence rather than individual component skills [1,2]. However, learners frequently struggle because competent execution of individual skills does not necessarily translate into coordinated performance under the cognitive, interpersonal and time pressures of authentic clinical encounters. Existing educational guidance commonly focuses on discrete examination tasks or individual competencies, offering limited support for diagnosing why integrated performance breaks down or how clinical teachers can target subsequent learning [3,4].

The 2023 revision of the Membership of the Royal Colleges of Physicians of the United Kingdom (MRCP (UK)) Practical Assessment of Clinical Examination Skills (PACES23) provides a useful example of this broader educational challenge. Implemented in 2023, PACES23 places greater emphasis on communication, consultation, shared decision-making and patient safety alongside physical examination and clinical reasoning, reflecting contemporary expectations of clinical practice [5-7]. Consequently, successful performance depends not simply on mastery of individual skills but on their coordinated integration within a single patient encounter [5].

PACES also has substantial international reach. In the Autumn 2025 examination diet, 3,002 candidates sat PACES, of whom 2,496 (83.1%) were international medical graduates and 1,613 (53.7%) sat the examination at international centres [8]. The examination is delivered through an established international network, making PACES23 relevant to postgraduate physicians and clinical educators well beyond the UK [9]. Although assessment structures differ internationally, the educational principles considered in this review may also have relevance to comparable high-stakes clinical performance assessments outside MRCP (UK), particularly where integrated clinical reasoning, communication and patient-centred care are assessed.

Although extensive literature exists on clinical reasoning, communication, feedback, simulation and workplace learning, these strands are often considered separately [10-15]. Practical frameworks are less well developed that help educators identify where integrated performance breaks down during clinical encounters and connect those observations with targeted educational responses. Such an approach could support both examination preparation and longitudinal workplace learning by moving beyond generic feedback towards diagnosis of specific performance gaps.

In this narrative educational review, we use PACES23 as an illustrative context to propose an integrated framework for teaching clinical performance. The framework couples a clinical enactment cycle (SEE → THINK → ACT), which identifies where performance breaks down during an encounter, with a complementary learning cycle (PRACTISE → PERFORM → REFLECT), which guides targeted educational responses. Operationalised through four educational domains and twelve evidence-informed strategies, the framework is intended to help clinical teachers translate observation into focused teaching and feedback. While developed using PACES23 as the exemplar, the framework may provide a useful conceptual approach for other high-stakes clinical performance assessments, although transfer beyond this context requires empirical evaluation.

## Review

Materials and methods

This article was developed as a structured narrative educational review rather than a formal systematic review. This design was chosen because the review question was integrative and practice-oriented: it required interpretation across heterogeneous sources, including official assessment guidance, conceptual scholarship, reviews and empirical studies, to connect PACES23 performance demands with educational mechanisms and observable teaching actions. The purpose was therefore explanatory and translational rather than estimation of a single intervention effect.

Literature was identified through targeted searches of PubMed/MEDLINE, ERIC and Google Scholar, alongside review of official MRCP (UK) PACES23 guidance and reference lists of relevant papers. Searches were conducted iteratively during manuscript development and updated to July 3, 2026. Search concepts included Practical Assessment of Clinical Examination Skills (PACES), Objective Structured Clinical Examinations (OSCEs), clinical skills assessment, postgraduate medical education, clinical reasoning, communication skills, feedback, simulation, deliberate practice, reflective learning, workplace learning and competency-based medical education. Priority was given to publications from 2000 onwards, while older landmark sources were retained where foundational to the educational concepts under discussion [1,2,10,11,16,17]. Searches were concept-led rather than designed for exhaustive retrieval; no Preferred Reporting Items for Systematic Reviews and Meta-Analyses (PRISMA)-style claim of systematic completeness is made.

Sources were selected where they directly informed the revised PACES23 format, provided relevant evidence from postgraduate clinical assessment or workplace-based medical education, or described educational theories with plausible applicability to bedside performance, communication, reasoning, feedback or patient safety. Preference was given to official guidance, influential conceptual papers, reviews and studies with direct practical relevance to postgraduate clinical training.

The synthesis proceeded in three stages. First, candidate and teaching behaviours were extracted from PACES23 guidance and the included literature and recorded against the educational problem they addressed [18]. Second, conceptually overlapping behaviours were merged, distinct behaviours retained and provisional strategies reviewed against all seven assessed PACES23 skills to check blueprint coverage. The twelve strategies were not pre-specified; they were the set retained after this iterative process. Strategy retention required each behaviour to satisfy three criteria: (1) identifiable relevance to at least one PACES23 assessed capability or integration need; (2) a plausible educational mechanism supported by relevant literature; and (3) translation into an observable candidate behaviour or clinical teacher action. Third, the retained strategies were grouped by principal educational function, producing four higher-order domains. The two-cycle framework was then developed as an integrative representation linking encounter-level enactment with longitudinal learning and refinement. Provisional groupings were repeatedly revisited against the source literature and PACES23 blueprint; categories were revised where they were redundant, insufficiently distinct or inadequately supported.

Educational theories were mapped to strategies by identifying the principal mechanism that most plausibly explained how each behaviour might support learning or performance. Where more than one mechanism was relevant, the dominant mechanism was retained for clarity, and supporting literature was cross-checked against the proposed teacher action. Theory was used as an explanatory lens rather than as evidence that any individual strategy has a PACES-specific causal effect. This distinction is important because much of the relevant evidence derives from broader health professions education rather than direct studies of PACES23.

The synthesis was shaped by the authors’ interpretive decisions about relevance, overlap, mechanism and practical teachability. The framework should therefore be understood as a transparent educational proposition rather than a value-neutral classification derived mechanically from the literature. To reduce unexamined inference, provisional categories were repeatedly checked against the PACES23 blueprint and supporting literature, explicit retention criteria were applied and the strategy-to-mechanism-to-teacher-action audit trail is reported in Table 1.

**Table 1:** Derivation and operationalisation of the integrated performance framework PACES23: Practical Assessment of Clinical Examination Skills 2023

Framework domain	PACES23 capability/identified need	Strategy	Principal educational mechanism/rationale	Operationalisation: candidate behaviour → teacher action	Indicative literature
Automate clinical performance	Physical examination; reproducibility under pressure	1. Reproducible examination routines	Deliberate practice; cognitive load	Candidate: rehearse systematic routines; teacher: observe a short sequence, give one targeted correction, repeat	[10,11,13,19]
	Physical signs; calibration of findings	2. Accurate sign elicitation	Experiential; workplace learning	Candidate: compare findings across real patients; teacher: ask trainee to demonstrate, describe and calibrate findings	[12,17,20,21]
	Clinical reasoning; common pattern recognition	3. Common clinical patterns	Pattern recognition; schema formation	Candidate: build illness scripts around common patterns; teacher: contrast similar cases and probe discriminating findings	[17,20,21]
	Integrated performance under realistic constraints	6. Timed realistic drills	Simulation; deliberate practice	Candidate: perform under realistic constraints; teacher: run brief timed encounters with focused debrief	[22,23,30,32,33]
Structure and externalise reasoning	Clinical reasoning; prioritisation under pressure	4. Rapid differential frameworks	Clinical reasoning; prioritisation	Candidate: prioritise likely and important diagnoses; teacher: ask for leading diagnosis, dangerous alternative and discriminators	[17,20,21]
	Communication of findings; concise synthesis	5. Concise presentation scaffolds	Cognitive load; communication structure	Candidate: present selective, synthesised findings; teacher: feedback on selection, synthesis and clarity	[7,13,19,20]
	Clinical reasoning; visible judgement	10. Reasoning out loud	Retrieval; reflective learning	Candidate: verbalise interpretation and judgement; teacher: use think-aloud prompts and identify unsupported assumptions	[14,24]
	Judgement; adaptation to new information	11. Follow-up questions	Retrieval; adaptive expertise	Candidate: connect findings to investigation and management; teacher: vary questions and introduce uncertainty or new information	[15,17,20,21]
Integrate communication and consultation	Communication; uncertainty and shared decisions	7. Deliberate communication rehearsal	Communication training; feedback	Candidate: explain, negotiate and respond to concerns; teacher: observe alignment between reasoning and explanation	[7,25,26]
	Consultation; integration of assessment and management	8. Complete consultation rehearsal	Integrated competence; workplace learning	Candidate: link assessment, explanation and management; teacher: use authentic encounters and feedback on transitions	[3,4,27]
Close the learning and safety loop	Longitudinal improvement; recurrent gaps	9. Recurrent weakness correction	Feedback; reflective practice	Candidate: track errors and set targeted goals; teacher: revisit the same goal in a later encounter	[14,25,26,28]
	Patient welfare; respect, comfort and safety	12. Maintain patient welfare throughout	Professional competence; patient-centred care	Candidate: treat patients respectfully, attend to comfort, preserve dignity and maintain safety; teacher: observe respect, comfort, dignity and safety throughout the encounter.	[3,5]

Results

PACES23 retains the five-station structure but redistributes emphasis across assessed competencies [5]. According to MRCP (UK) guidance, Stations 1 and 4 place substantial emphasis on communication alongside focused physical examination, while Stations 2 and 5 focus more heavily on consultation and clinical management. Physical examination remains central, especially in Stations 1, 3 and 4, but is assessed as part of integrated clinical performance rather than as an isolated technical task [5].

**Figure 1:**
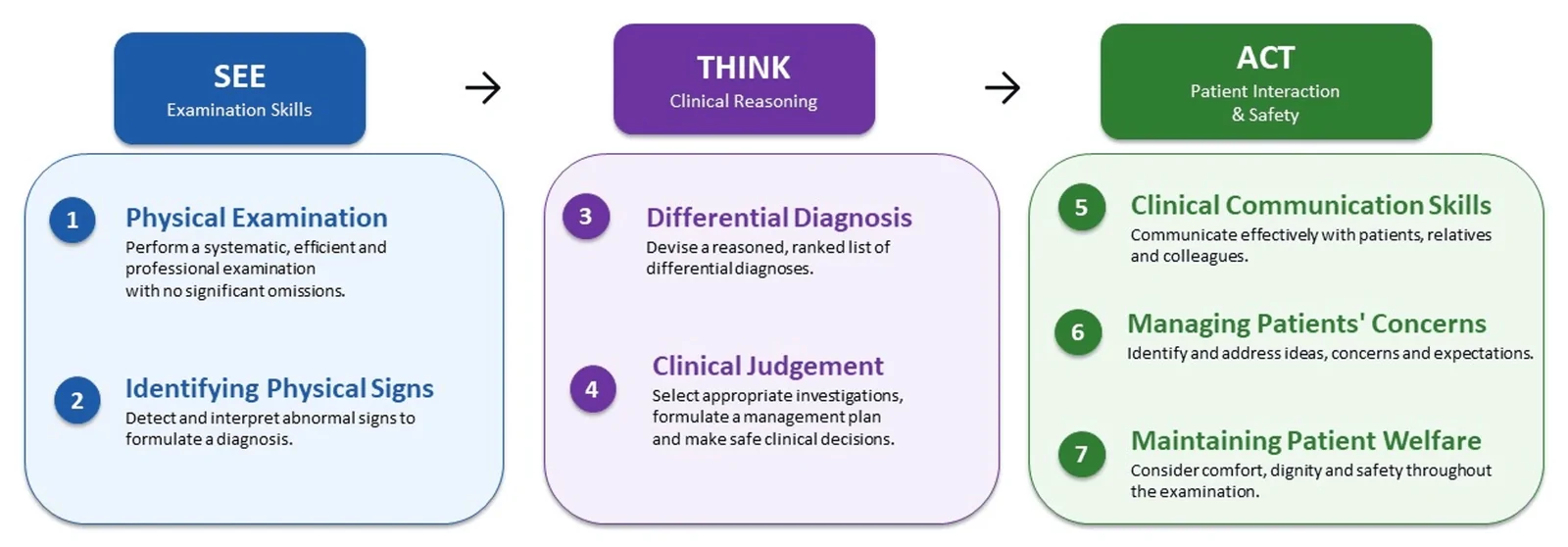
PACES23 assessed capabilities organised as SEE → THINK → ACT Author-created synthesis based on publicly available MRCP (UK) PACES23 guidance. SEE comprises physical examination and identification of physical signs; THINK comprises clinical reasoning and clinical judgement; ACT comprises communication, management of patients’ concerns and maintenance of patient welfare. This is a pedagogic memory map, not an official MRCP (UK) classification. PACES23: Practical Assessment of Clinical Examination Skills 2023, MRCP (UK): Membership of the Royal Colleges of Physicians of the United Kingdom

The seven core skills assessed include physical examination, identification of physical signs, clinical communication, differential diagnoses, clinical judgement, management of patients’ concerns and maintenance of patient welfare [5]. These domains are sampled across the examination rather than being confined to single stations, supporting judgements based on performance across contexts [2,3]. Figure 1 reorganises the seven assessed skills as SEE → THINK → ACT. This is an author-created teaching and memory aid rather than an official MRCP (UK) classification: its purpose is to help candidates recall which capabilities are being assessed and where they sit within the encounter-level performance sequence.

From an educational perspective, the revised format is consistent with broader moves towards assessment of integrated competence and observable performance [2,4,19,23]. The practical implication for teachers is that communication and consultation should not be treated as secondary or rehearsed only in designated communication sessions. Examination, reasoning, explanation, management and patient-centredness need to be developed together.

Several educational principles help explain how integrated clinical performance may be developed. Deliberate practice emphasises focused repetition, clear goals and corrective feedback [10,11]. Cognitive load theory offers one explanation for why reproducible routines may be useful under time pressure: when lower-level processes are well rehearsed, limited cognitive resources may be more available for interpretation, prioritisation and interaction [13,28].

Experiential and reflective learning emphasise cycles of encounter, reflection, refinement and re-application [12,14,25]. Feedback is central because recurrent weaknesses may persist unless they are made visible and addressed through targeted practice [20,25,26]. Clinical reasoning literature supports the use of structured differentials, concise summaries and explicit justification of management decisions [17,21,29,30]. Competency-based perspectives further emphasise observable, safe and trustworthy performance in authentic contexts [3,4,16,19]. Contemporary scholarship also highlights the context-dependent use of workplace-based assessment, feedback literacy, longitudinal assessment and explicit clinical reasoning instruction as mechanisms for turning observed performance into learning [15,22-24,27,31].

These theories are not presented as direct validation of the proposed framework. Rather, they provide complementary explanatory mechanisms for why particular preparation and teaching behaviours may support integrated performance.

**Figure 2:**
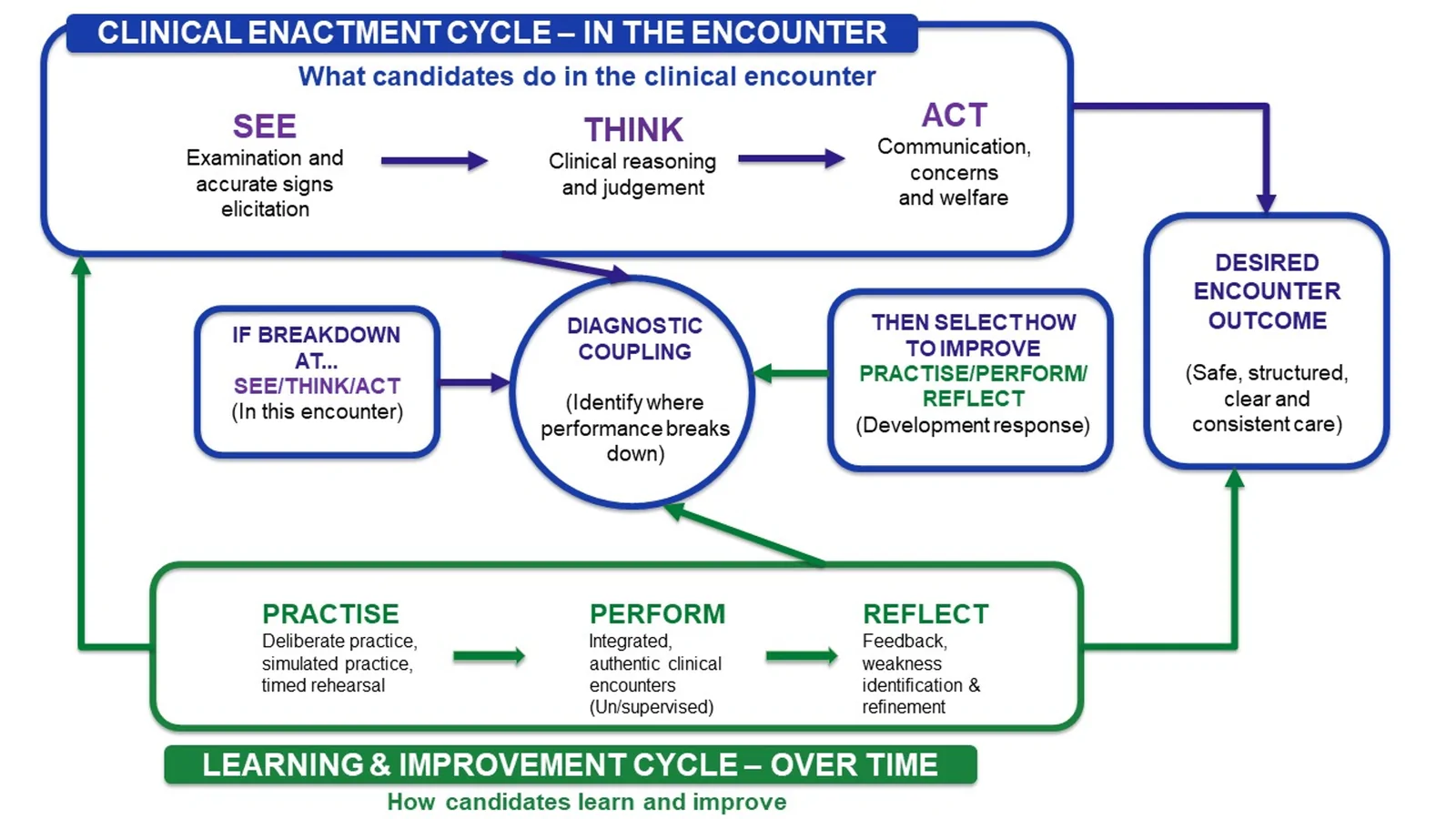
Integrated performance framework for PACES23 Diagnostic coupling links the clinical enactment cycle (SEE → THINK → ACT), which locates encounter-level breakdown, with the learning and improvement cycle (PRACTISE → PERFORM → REFLECT), which guides the targeted developmental response. The learning loop is intended to improve subsequent encounters. PACES23: Practical Assessment of Clinical Examination Skills 2023

We propose that integrated PACES23 performance can be understood through two complementary cycles. The clinical enactment cycle, SEE → THINK → ACT, represents systematic examination and accurate sign elicitation (SEE), interpretation and prioritised clinical reasoning (THINK), and communication, management and patient safety (ACT). This helps identify distinct causes of performance breakdown requiring different educational responses. The learning cycle, PRACTISE → PERFORM → REFLECT, addresses these gaps through deliberate rehearsal, authentic integrated performance, and feedback-informed reflection and refinement. Together, the cycles link encounter-level diagnosis with longitudinal development, aiming for safe, structured, clear and consistent clinical performance (Figure 2).

The twelve strategies were organised into four higher-order domains according to their principal educational function: (1) automate clinical performance, (2) structure and externalise reasoning, (3) integrate communication and consultation and (4) close the learning and safety loop. The individual strategies are described below, while Table 2 provides a concise, candidate-facing quick-reference guide designed to facilitate their practical use during PACES23 preparation.

**Table 2:** Candidate-facing quick-reference guide translating the framework into a portable practical checklist for PACES23 preparation PACES23: Practical Assessment of Clinical Examination Skills 2023

No.	PACES23 practice recommendation
1	Develop reproducible examination routines
2	Prioritise accurate elicitation of signs
3	Build familiarity with common clinical patterns
4	Build rapid differential frameworks
5	Use concise presentation scaffolds
6	Practise timed drills under realistic conditions
7	Rehearse communication deliberately
8	Rehearse complete consultation and management encounters
9	Identify and correct recurrent weaknesses through feedback
10	Practise reasoning out loud
11	Anticipate clinically relevant follow-up questions
12	Maintain patient welfare throughout

Develop Reproducible Examination Routines

Core examination routines should become systematic and reproducible. Focused repetition with feedback may reduce avoidable cognitive burden, allowing greater attention to interpretation and patient interaction [10,11,13,29]. Practice should emphasise consistency rather than rigid choreography.

Prioritise Accurate Elicitation of Signs

PACES remains grounded in bedside examination, and inaccurate elicitation of a major sign can distort subsequent reasoning. Repeated exposure to real patients, comparison across cases and calibration with experienced clinicians may improve accuracy [12,17,21].

Build Familiarity With Common Clinical Patterns

Preparation should prioritise repeated exposure to common and clinically important patterns rather than unusual diagnoses. Pattern recognition and schema development are established components of clinical reasoning expertise [17,21], particularly when similar presentations are deliberately contrasted.

Build Rapid Differential Frameworks

Structured differentials can help candidates prioritise likely and important diagnoses under pressure [17,21]. The aim is not exhaustive recall but defensible prioritisation, including recognition of dangerous alternatives and discriminating findings.

Use Concise Presentation Scaffolds

Short, structured presentations may reduce omission and support clarity under cognitive load [7,13,17,29]. The scaffold should support rather than replace patient-specific reasoning. Teachers can limit presentations to a defined time and provide feedback separately on content selection, synthesis and clarity.

Practise Timed Drills Under Realistic Conditions

PACES performance is constrained by time, stress and multitasking. Simulation and mock encounters can approximate these demands and support deliberate rehearsal [30,32,33]. Brief timed practice may be embedded throughout preparation rather than reserved for full mock examinations.

Rehearse Communication Deliberately

Communication skills develop through performance, observation and feedback rather than reading alone [7,20,27]. Rehearsal should include explanation, uncertainty, shared decision-making and difficult conversations, while maintaining alignment between clinical reasoning and what is communicated.

Rehearse Complete Consultation and Management Encounters

Consultation requires integration of information gathering, reasoning, decision-making and communication. Preparation is therefore strengthened when candidates rehearse complete scenarios rather than fragmented subskills [4,19,34]. Teachers can use authentic outpatient or ward encounters and provide feedback on transitions between assessment, explanation and management planning.

Identify and Correct Recurrent Weaknesses Through Feedback

Feedback is most useful when it makes a specific performance gap visible and informs subsequent action [20,27]. Recurrent errors should be converted into targeted practice goals and revisited in later encounters, closing the feedback loop [14,26].

Practise Reasoning Out Loud

Verbalising interpretation and judgement can expose hesitation, unsupported assumptions and gaps that remain hidden during silent study [26,35]. The aim is selective externalisation of clinically relevant reasoning rather than continuous narration.

Anticipate Clinically Relevant Follow-Up Questions

Questions about differentials, investigations, management and prioritisation may support retrieval, structured reasoning and adaptation to new information [17,21,36]. Variation and uncertainty are important because expertise requires adjustment rather than reproduction of fixed answers.

Maintain Patient Welfare Throughout

Patient welfare is a continuously assessed domain in PACES23, requiring candidates to treat patients respectfully while ensuring their comfort and safety [4,5,37]. Candidates should demonstrate respectful interaction, attend to discomfort, preserve dignity and recognise actions needed to maintain safety throughout the encounter. These behaviours should be integrated into clinical performance rather than treated as a final safety check.

This can be illustrated with an example. During a timed cardiovascular encounter, a trainee incompletely elicits a key murmur and associated signs (SEE), offers a non-prioritised differential without identifying a dangerous alternative (THINK) and does not clearly explain the need for escalation (ACT). Observation during PERFORM allows the teacher to locate these breakdowns rather than giving generic feedback. The trainee then undertakes focused PRACTISE: repeated sign elicitation with calibration, brief differential-prioritisation drills and explicit escalation rehearsal. In REFLECT, the teacher and trainee identify which errors recur, set one or two specific goals and reassess them in a subsequent authentic encounter. The example illustrates how the framework can connect diagnosis of a performance gap with a targeted educational response.

Discussion

The revision of MRCP (UK) PACES reflects wider developments in postgraduate medical education, where assessment increasingly emphasises integrated clinical competence rather than isolated technical skill. Contemporary physicians are expected to recognise physical signs, formulate and prioritise differentials, communicate clearly, involve patients in decisions and maintain safety [4-6,19]. Preparation centred mainly on memorising rare signs or rehearsing examination technique in isolation is therefore incompletely aligned with the competencies sampled in PACES23.

The principal conceptual contribution proposed here is the diagnostic coupling of encounter-level performance with longitudinal educational response [10-12,14,15,20,22-28,30,32-34]. Rather than relabelling established theories, the framework makes them more actionable by linking an observed SEE, THINK or ACT breakdown to focused practice, authentic performance, structured reflection or a combination.

Existing educational theories, including deliberate practice, cognitive load theory, experiential learning and competency-based medical education, provide complementary explanations for how clinicians develop expertise and how learning can be supported. Our contribution is not to propose another learning theory but to translate these established concepts into a practical diagnostic framework that links observed performance breakdown with targeted educational responses during workplace teaching. In doing so, the framework connects observation, diagnosis of performance gaps and subsequent learning, providing clinical teachers with an actionable educational approach [3,4,12-14,16,19,26,29].

This coupling helps teachers move between component improvement and whole-task integration without assuming that either isolated practice or repeated whole-task performance is sufficient.

The four domains translate the framework into teachable activity by locating problems within reproducibility and accuracy, visible reasoning, integrated consultation or feedback and patient welfare. They are intended as a practical diagnostic aid rather than a new taxonomy of competence.

The framework is suited to workplace teaching because these capabilities can be developed through ordinary clinical work, not only dedicated examination courses. A ward round can pair focused examination with a prioritised differential; an outpatient encounter can expose transitions from explanation to shared planning and a workplace-based assessment can identify one recurrent gap for later reassessment. The teacher thereby facilitates integrated performance development rather than supplying examination tips.

The framework should primarily be viewed as an educational model developed within the context of PACES23. Its transferability to other assessment systems requires empirical evaluation. Its value should be judged by whether it helps candidates and teachers organise assessed skills, diagnose performance problems and select targeted responses. Future work should evaluate whether use of the framework improves feedback quality, learner performance and transfer to workplace practice across different clinical training environments.

For clinical teachers, the framework suggests a shift from episodic ‘PACES teaching’ towards repeated observation of integrated performance. Short episodes can target timed examination, prioritised differentials, explanation or escalation, followed by a specific practice goal and later reassessment. Feedback should identify the breakdown and what the trainee should do differently next time.

For programmes, the four domains offer a curriculum structure linking bedside examination, reasoning aloud, structured presentation, communication rehearsal, complete consultations and explicit attention to patient welfare. Faculty development can focus on observing integration and converting feedback into subsequent practice [4,17,19].

Limitations

This narrative educational review used a targeted rather than exhaustive search, so relevant literature may have been missed. Formal systematic review screening and study-level risk-of-bias assessment were not undertaken because of the narrative design and heterogeneous source types. Strategy selection, domain construction and framework development involved author judgement. Much of the supporting evidence derives from broader health professions education rather than PACES23-specific studies. The proposed mechanisms should therefore be viewed as plausible educational interpretations rather than empirically validated effects.

## Conclusions

PACES23 demands coordinated clinical performance under time pressure. This review translates that challenge into a practical approach for learning, teaching and feedback. Four domains and twelve evidence-informed strategies connect assessed capabilities with observable candidate behaviours and teacher actions. The framework may help candidates structure their preparation and clinical teachers move beyond generic feedback by identifying specific gaps and selecting focused educational responses. Its aim is to support safer, clearer and more consistent performance through longitudinal workplace learning rather than short-term examination coaching. Future empirical studies should evaluate its educational impact, transferability and effectiveness across different clinical training settings.

## Appendices

Supplementary material 1: Search strategy

Table 3 presents the search strategy used in the present study.

**Table 3:** Search strategy

Database	Search concepts/strategy	Last updated
PubMed/MEDLINE	(PACES OR "Practical Assessment of Clinical Examination Skills" OR OSCE OR "clinical skills assessment") AND ("clinical reasoning" OR communication OR feedback OR simulation OR "deliberate practice" OR "reflective learning" OR "workplace learning" OR "competency-based medical education")	July 3, 2026
ERIC	("PACES" OR "Practical Assessment of Clinical Examination Skills" OR "OSCE" OR "objective structured clinical examination" OR "clinical skills assessment") AND ("clinical reasoning" OR "communication skills" OR "feedback" OR "simulation" OR "deliberate practice" OR "reflective learning" OR "workplace learning" OR "competency-based medical education")	July 3, 2026
Google Scholar	Separate broad searches combining “PACES23”, “MRCP PACES”, “OSCE” or “clinical skills assessment” with one or more of the following concepts: “clinical reasoning”, “communication”, “feedback”, “simulation”, “deliberate practice”, “reflective learning”, “workplace learning” and “competency-based medical education”.	July 3, 2026
Other sources	Official MRCP (UK) PACES23 guidance and backward reference searching	July 3, 2026
